# The Time to Spontaneous Drop of Polyglactin 910 (Vicryl) in the Nasal Cavity

**DOI:** 10.7759/cureus.62335

**Published:** 2024-06-13

**Authors:** Tomotaka Hemmi, Kazuhiro Nomura, Kazuhiro Omura, Teppei Takeda, Mitsuru Sugawara, Ryoukichi Ikeda

**Affiliations:** 1 Otolaryngology, Tohoku Kosai Hospital, Sendai, JPN; 2 Otolaryngology, Head and Neck Surgery, The Jikei University School of Medicine, Tokyo, JPN; 3 Otolaryngology, Head and Neck Surgery, Iwate Medical University, Yahaba, JPN

**Keywords:** treatment, pain, suture, endoscopic sinus surgery, surgery

## Abstract

Sutures play a crucial role in closing mucosal incisions during endoscopic nasal surgery. The duration until the spontaneous drop of polyglactin 910 (Vicryl) sutures in the nasal cavity remains uncertain. To investigate this, we examined the medical records of patients who underwent septoplasty, inferior turbinate reduction, or endoscopic modified medial maxillectomy with polyglactin 910 sutures. The sutures were counted and monitored during follow-up visits, and removal occurred only if patients reported discomfort. In our study of 124 patients, a total of 453 sutures were placed during surgery. Eighteen sutures had to be intentionally removed due to discomfort. Importantly, no surgical site infections were observed during the follow-up period. We found that sutures on the lateral nasal wall persisted longer than those on the nasal septum, with respective half-lives of 70 days and 64 days (p = 0.0071). In conclusion, using polyglactin 910 sutures in nasal surgery and allowing them to dissolve naturally in the submucosa is an effective approach. The sutures exhibit longer persistence on the lateral nasal wall compared to the nasal septum.

## Introduction

Sutures play a crucial role in closing mucosal incisions during septoplasty procedures, anterior lateral nasal wall incisions for inferior turbinate reduction, and endoscopic modified medial maxillectomy (EMMM) [1-3]. Surgeons choose suture materials based on their preferences, balancing options for absorbability (absorbable versus nonabsorbable) and suture patterns (monofilament versus braided) [4-6]. For instance, Ethilon, a nonabsorbable monofilament nylon suture, offers benefits such as reduced infection risk and minimal pain during removal but can pose challenges due to its rigidity, lower knot strength, and sharp tips left in knots [4]. Conversely, Vicryl, an absorbable braided suture made from polyglactin 910, provides pliability and flexibility for ease of handling but may lead to infection or pain during removal if necessary.

Polyglactin 910, an absorbable suture, has an absorption time of 56 to 70 days in the body. Due to this long duration, the suture may need to be removed one to two weeks after surgery [4, 6, 7]. However, in our practice, we use polyglactin 910 for suturing mucosal incisions in the nasal cavity, allowing the suture to dissolve naturally without complications. In this study, we reviewed medical records and assessed suture survival rates across different sites to provide patients with information on the expected time for suture spontaneous drop.

## Materials and methods

This study was conducted at Tohoku Kosai Hospital in Japan from January 2023 to August 2023. Patients who underwent septoplasty, inferior turbinate reduction, or EMMM, and had mucosal incisions sutured with polyglactin 910 (5-0 Coated Vicryl with TF needle 13 mm, 1/2c, Ethicon Inc., Somerville, New Jersey) were included in the evaluation. We applied gentamicin ointment (Gentamicin Sulfate Ointment 0.1% "IWAKI", Iwaki Seiyaku Co., Ltd., Tokyo, Japan) to polyglactin 910 sutures to enhance their smoothness. The study received approval from the institutional review board (kkrtohoku-202312otor_S1-1_01), and all procedures adhered to the ethical standards established by the Helsinki Declaration of 1975, as revised in 1983.

The surgical approach for septoplasty utilized either a Killian incision or a hemitransfixion incision, based on the presence of caudal deviation. A 1 mm silicon sheet, tailored according to a prior study [1], was applied to gently stabilize the nasal septum. The sheet was kept in place for one to two weeks in patients with a Killian incision and for two to four weeks in patients with a hemitransfixion incision [8].

For the inferior turbinate reduction, the procedure adhered to a previously described technique [2]. In short, a long mucosal incision was made at the mucocutaneous junction with a 15c scalpel blade, allowing access to the entire inferior turbinate. A submucosal space was then created using the 15c scalpel blade to visualize the submucosal tissue. A microdebrider specifically designed for turbinate surgery (2 mm inferior turbinate blade, #1882040HR, Medtronic) was used to resect the submucosal tissue. After completing the submucosal resection, the turbinate bone was gently displaced laterally with an elevator within the mucosa, preserving the mucosal surface. The redundant mucosa was trimmed, and the incision was sutured with polyglactin 910. In the case of EMMM, a long incision was made at the piriform aperture [3, 9]. After inferior turbinate reduction or EMMM, the incision at the lateral nasal wall was covered using Plus Moist HS-W, a dissolvable calcium alginate dressing [10, 11]. Postoperatively, patients were instructed to rinse their noses with saline solution twice daily starting two days after surgery.

The number of remaining sutures was counted and recorded during each follow-up outpatient visit. The sutures were not removed unless patients reported discomfort related to them.

The survival of the sutures was visualized using Kaplan-Meier graphs, comparing suture survival between the nasal septum and lateral nasal wall. The Wilcoxon test was used to evaluate differences in suture survival between these two areas. Factors such as sex, age (older age defined as ≥ 60 years), and type of incision (Killian or hemitransfixion for the nasal septum, and inferior turbinate reduction or EMMM for the lateral nasal wall) were analyzed for their association with suture survival using the Wilcoxon test. Analyses were performed with Stata version 14.0 (Stata Statistical Software, College Station, Texas), with a p-value < 0.05 considered statistically significant.

## Results

A total of 124 patients participated in the study, with their baseline characteristics summarized in Table 1.

**Table 1:** Patients' background EMMM, endoscopic modified medial maxillectomy.

Variable	Number
Sex	
Male	93
Female	31
Age (years)	
Minimum	17
Maximum	79
Average	47
≥60 years	28
<60 years	96
Suture site	
Nasal septum	
Killian incision	78
Hemitransfixion incision	32
Lateral nasal wall	
Inferior turbinectomy	99
EMMM	12
Number of sutures	
Nasal septum	303
Lateral nasal wall	150

**Figure 1:**
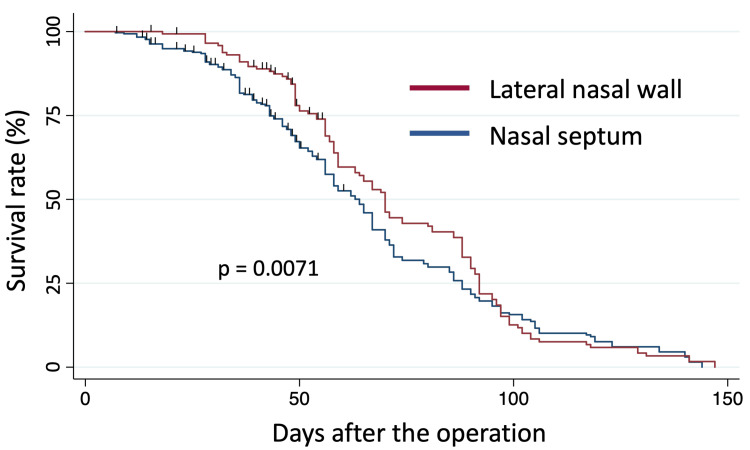
The Kaplan-Meier survival curve The Kaplan-Meier survival curve for Polyglactin 910 (Vicryl) sutures, categorized by suture site.

At the conclusion of surgery, 453 sutures were placed. Sutures were intentionally removed in 18 patients. During follow-up visits, no surgical site infections were observed. The survival curve of sutures is illustrated in Figure 1.

Sutures at the lateral nasal wall tended to last longer than those at the nasal septum (p = 0.0071). The suture survival rates at postoperative days 30, 60, 90, 120, and 150 are summarized in Table 2.

**Table 2:** Survival rates of sutures

	Nasal septum		Lateral nasal wall	
Post-operative days	Survival rate	95% CI	Survival rate	95% CI
30	90%	86%-93%	96%	91%-98%
60	53%	46%-59%	58%	49%-66%
90	22%	16%-28%	29%	22%-38%
120	6%	3%-10%	4%	2%-9%
150	0%		0%	

The half-life of sutures was 64 days on the nasal septum and 70 days on the lateral nasal wall. Factors such as sex (nasal septum p = 0.2040, lateral nasal wall p = 0.1704), age (older age defined as ≥60 years) (nasal septum p = 0.6465, lateral nasal wall p = 0.9020), and type of incision (Killian or hemitransfixion for the nasal septum (p = 0.3190), and inferior turbinate reduction or EMMM for the lateral nasal wall (p = 0.7914)) did not significantly affect the survival of sutures. 

## Discussion

Our investigation found that polyglactin 910 sutures can remain in the nasal cavity for up to three months, with sutures at the lateral nasal wall lasting longer than those at the nasal septum. No factors were found to influence the duration until the sutures naturally dropped out.

The sutures at the lateral nasal wall remained in place longer than those at the nasal septum (Table 2, Figure 1). This may be because the submucosal tissue is thicker at the lateral nasal wall, allowing sutures to be placed deeper and thus be more secure. However, the time until spontaneous suture dropout did not differ significantly between the Killian incision and the hemitransfixion incision. The hemitransfixion incision is located at the caudal end of the septal cartilage, where the mucosa transitions to skin, potentially providing more secure suturing than the Killian incision, which is situated about 10 mm cranial.

The incisions for inferior turbinate reduction and EMMM are on nearly the same line [2, 3, 9]. The main distinction between these two procedures lies in the length of the incision: the EMMM incision extends to the nasal floor. Given this, it is understandable that there was no significant difference in suture survival between the inferior turbinate reduction and EMMM procedures.

The suture materials used in the nasal cavity differ in their effects on crust formation and bacterial colonization [12]. Encrustation is more frequently observed with polyglactin 910 and irradiated polyglactin 910 compared to nonabsorbable monofilament polypropylene sutures and absorbable monofilament polyglytone 6211 sutures. Additionally, the number of colony-forming units per centimeter was higher in braided sutures. In our study, while the frequency of crusting was not recorded, it was occasionally observed. Notably, 18 out of 124 patients reported discomfort due to crusting or itching and subsequently had their sutures removed.

Aging and sex differences did not influence the duration of suture retention. The mucosal epithelium of the middle turbinate tends to thin with age [13], and intranasal volume increases as people get older [14]. However, these age-related changes may not be substantial enough to affect suture stability. Additionally, the thickness of the inferior turbinate does not vary significantly between sexes [15].

Polyglactin 910 sutures can remain in the nasal cavity for up to three months (Table 2), which is longer than the typical necessary period of about one week. The sutures lose total tensile strength 28 days after surgery and fully absorb within 56 to 70 days [6]. A modified version, irradiated polyglactin 910 (Vicryl Rapide, Ethicon Inc., Somerville, New Jersey), loses its total tensile strength at 14 days and fully absorbs within 40 to 50 days. It is used for skin grafts and spontaneously dissolves 7 to 10 days after surgery [7].

However, not all types of needles available for polyglactin 910 are available for irradiated polyglactin 910, especially the needle type we prefer to use. In the confined space of the nasal cavity, needle options are limited. Among a series of 124 patients, 18 reported discomfort or itching around the suture site and had their sutures removed, while the rest did not experience discomfort and allowed the sutures to dissolve naturally. By avoiding premature removal, patients can avoid pain and discomfort associated with the suture removal. It is advisable to ask patients if the sutures are causing any issues and, if not, to leave them untouched for natural absorption.

There are some limitations in this study. The actual timing of suture dropout is earlier than the data suggest because postoperative days were measured according to the day of the visit. Initially, patients visit the hospital every one to two weeks, but later the visits are spaced out to every one to two months. For example, if a patient comes in two weeks after surgery (postoperative day 14) and the sutures are still intact, but the sutures drop out the next day (postoperative day 15), and the patient returns four weeks after surgery (postoperative day 28), the data record the dropout as occurring on postoperative day 28, even though it actually happened on day 15. The exact dropout date cannot be determined without examining the patient daily. As time passes, this discrepancy increases due to the longer intervals between visits, resulting in an overestimation of the actual dropout day. We used the Wilcoxon test to compare suture survival between groups. The Wilcoxon test is generally more sensitive to detecting early differences in survival than the log-rank test [16].

## Conclusions

Polyglactin 910 sutures spontaneously drop within three months without major complications. No surgical site infections were observed. While crusting did occur on the sutures, most patients did not experience any discomfort. However, 18 out of 124 patients reported discomfort due to crusting or itching and subsequently had their sutures removed.

Using polyglactin 910 sutures in the nasal cavity and allowing them to naturally absorb unless patients report discomfort is an effective option. If irradiated polyglactin 910 sutures with an appropriate needle size are available, they provide an even better alternative due to their faster absorption rate.
